# Mowing strategies for controlling *Cirsium arvense* in a permanent pasture in New Zealand compared using a matrix model

**DOI:** 10.1002/ece3.2090

**Published:** 2016-03-28

**Authors:** Graeme W. Bourdôt, Britta Basse, Michael G. Cripps

**Affiliations:** ^1^ AgResearch LimitedPrivate Bag 4749Christchurch8140New Zealand

**Keywords:** Californian thistle, Canada thistle, creeping thistle, management, mathematical model, mowing, perennial thistle

## Abstract

Defoliation has frequently been proposed as a means of controlling *Cirsium arvense* (L.) Scop. (Californian thistle, Canada thistle, creeping thistle, perennial thistle), an economically damaging pastoral weed in temperate regions of the world, but its optimization has remained obscure. We developed a matrix model for the population dynamics of *C. arvense* in sheep‐grazed pasture in New Zealand that accounts for the effects of aerial shoot defoliation on a population's photosynthetic opportunity and consequential overwintered root biomass, enabling mowing regimes varying in the seasonal timing and frequency of defoliation to be compared. The model showed that the long‐term population dynamics of the weed is influenced by both the timing and frequency of mowing; a single‐yearly mowing, regardless of time of year, resulted in stasis or population growth, while in contrast, 14 of 21 possible twice‐yearly monthly mowing regimes, mainly those with mowing in late spring, summer, and early autumn, resulted in population decline. Population decline was greatest (with population density halving each year) with twice‐yearly mowing either in late spring and late summer, early summer and late summer, or early summer and early autumn. Our results indicate that mowing can be effective in reducing populations of *C. arvense* in pasture in the long term if conducted twice each year when the initial mowing is conducted in mid spring followed by a subsequent mowing from mid summer to early autumn. These mowing regimes reduce the photosynthetic opportunity of the *C. arvense* population and hence its ability to form the overwintering creeping roots upon which population growth depends.

## Introduction

**Figure 1:**
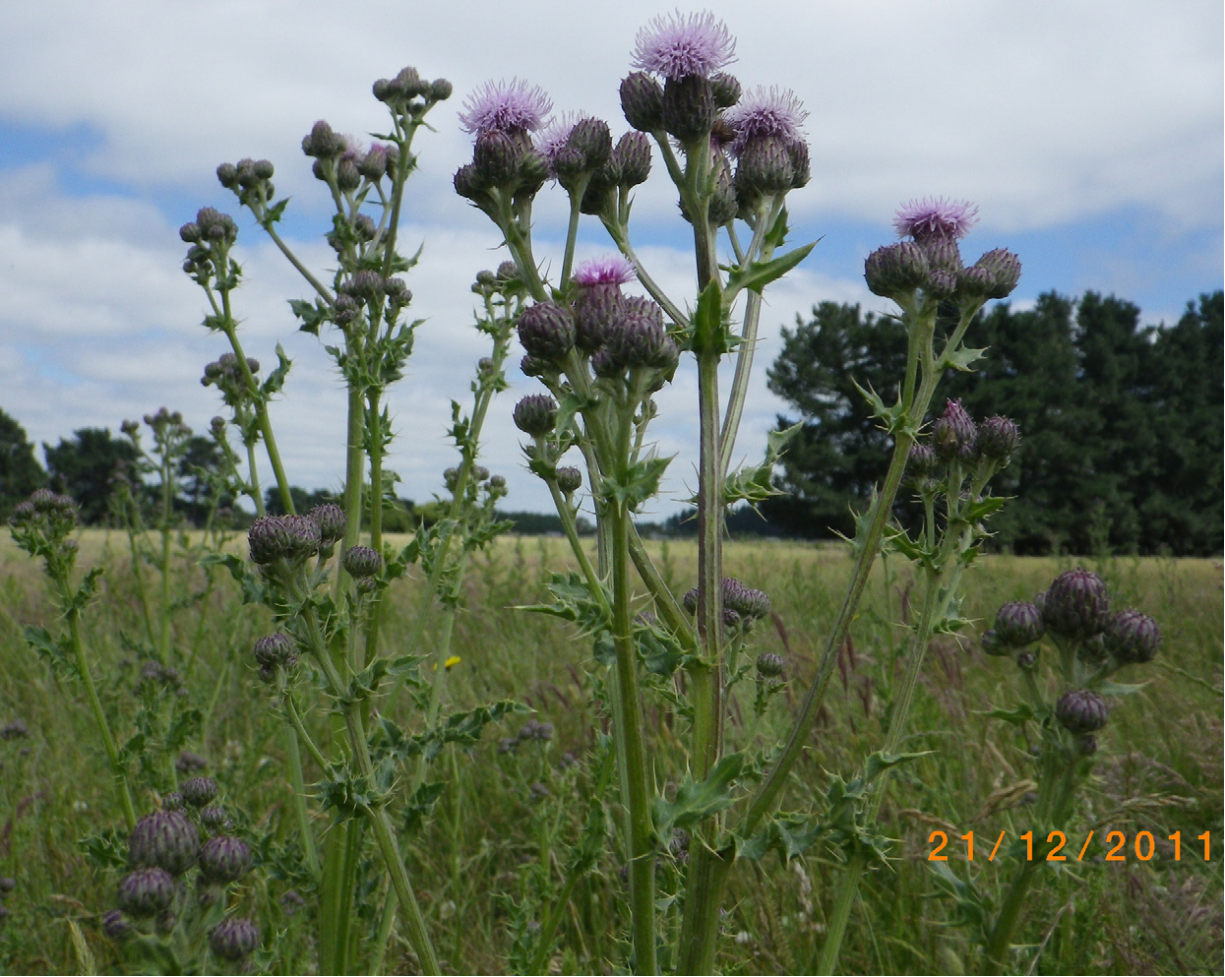
An infestation of *Cirsium arvense* in a pasture in New Zealand. A male plant of this dioecious species is shown in the foreground.

Mowing pastures infested by *Cirsium arvense* (L.) Scop. (Californian thistle, Canada thistle, creeping thistle, perennial thistle) (Fig. 1) has long been considered a potentially effective control method for this persistent and economically damaging perennial weed in both its native European and exotic ranges (Canada, USA, Australia, New Zealand). In an essay, “Extirpation of Canada Thistles” published in the Transactions of the New‐York State Agricultural Society in 1847, the author lamented that 57 years after the state of Vermont passed a law directing the weed's destruction (in 1795), “As yet no one has thought of generalizing the accumulated facts of past experience …”. The author went on to deduce from the work of others that “grasses [sown] will be found the easiest [but slow] means of destruction”, that “….by the plow, hoe, fire or salt [on shallow soils], the thistle may be [rapidly] destroyed” and that “Mowing [when the plant is in bloom] will destroy those parts of the thistle which have thrown up flowering stalks” (Stevens 1847). During the 168 years since this early synthesis, many field experiments have provided substantial evidence that mowing can be effective in reducing *C. arvense* shoot populations in pastures. They show that the frequency and timing of the defoliation within a growing season, and the number of consecutive years in which it is repeated, all influence the rate of population decline, but their optimal combination remains obscure.

A single mowing to ground level in early‐ to mid‐summer, when many of the shoots would have been flowering, caused population decline in *C. arvense* in pastures in Australia (Amor and Harris 1977) and in New Zealand (Mitchell and Abernethy 1993, 1995), and in roadside vegetation in England (Parr and Way 2005). However, two mowing events caused a more rapid decline than one mowing in the Australian pasture (Amor and Harris 1977). Additional mowing events within a growing season may further increase the rate of population decline as was found in a pasture in Colorado, USA, where three mowing events during the growing season resulted in a more rapid decline than two (Beck and Sebastian 2000). Mowing on three occasions in a grassland in England (Hedworth‐Foulkes 1909), on four occasions in the USA (Schreiber 1967), and on five occasions in roadside vegetation in England (Parr and Way 2005) all resulted in population declines in *C. arvense*. However, as other mowing frequencies were not included in these experiments, the extent to which these additional defoliation events would have increased the rate of decline over that with one or two events is unknown.

The mowing treatments in these experiments were widely distributed across almost all months of the growing season from early spring (March and September in the northern and southern hemispheres, respectively) through until late autumn (November and May in the northern and southern hemispheres, respectively). In addition to this variation in the number and timing of mowing treatments, the experiments have varied greatly in the number of consecutive years during which the mowing treatments were applied; from two years (Hedworth‐Foulkes 1909; Amor and Harris 1977; Mitchell and Abernethy 1993, 1995; Hurrell and Bourdôt 1996; Beck and Sebastian 2000) to four (Schreiber 1967) and up to eighteen years (Parr and Way 2005). These experiments clearly confirm that defoliation of *C. arvense* on one or more occasions during the period of the year when the aerial shoots are present (the growing season) can result in a negative rate of growth in the aerial shoot population and hence population decline. But, due to the practical limitations of field experiments, none encompassed all possible combinations of frequency and timing of mowing and so their optimal combination for a desired rate of population decline remains unknown.

An alternative approach to defining optimal defoliation regimes for managing *C. arvense* in a permanent grassland, and perhaps coming closer to meeting Stevens's challenge of “generalizing the accumulated facts of past experience” (Stevens 1847), is to develop a mechanistic model. Such a model would ideally provide a tractable mathematical framework that might not only explain the population dynamics of *C. arvense* in a grassland system, but also, by accounting for current understanding of the phenological and physiological characteristics of growth in biomass of the plant, facilitate prospective analysis of a wide range of mowing strategies differing in the frequency and timing of defoliation. Models to date for the population dynamics of *C. arvense* have been either too simplistic or too coarse in their handling of within‐season transitions for this purpose (Forsyth 1985; Blumenthal and Jordan 2001; Chalak et al. 2008) or have been restricted to a specific life‐history stage such as shoots and the timing of their emergence (Donald 2000).

Shea and Kelly (1998) suggest that matrix models (Caswell 2001) can help elucidate the life‐history stage of a weed that should be targeted for control and enable quantitative comparison of the impacts of alternative control options on population size over the long term. Here, we use the conceptual framework for the population dynamics of *C. arvense* outlined by Leathwick and Bourdôt (2012) to develop a matrix model for the species in a sheep‐grazed pasture. The model is based on current knowledge, much of which is discussed in a variety of reviews (Hansen 1918; Haggar et al. 1986; Nadeau and Vanden Born 1989; Bond et al. 2007; Tiley 2010). Central to the model is a linear relationship between the accumulated biomass of the weed's propagative bud‐bearing creeping roots, the life‐history stage widely recognized as driving the weed's persistence (Andersson et al. 2013) and the population's photosynthetic opportunity during the growing season (Bourdôt et al. 1998). The model uniquely accounts for the ephemeral nature of the propagative roots of *C. arvense* (Rogers 1928; Sagar and Rawson 1964; Bourdôt et al. 2000) and the absolute dependence in a pasture of each year's adventitious shoot population on the mass of overwintered root and its attendant buds (Bourdôt et al. 2006). We use the model to compare mowing regimes with either one or two events per year on the basis of their modeled stable‐state population growth rate projections.

## Materials and Methods

Three key steps were taken to explore the response of a *C. arvense* population in sheep pasture to alternative mowing regimes. First, we developed a one‐year model that “grows” an aerial shoot population solely from an overwintered creeping root population; seeds were assumed to make no contribution to the shoot population (Leathwick and Bourdôt 2012). Second, we extended this model to multiple years, and third, we used this multiple‐year model to simulate a variety of mowing regimes to assess the long‐term effectiveness of defoliation by mowing as a control method.

### One‐year model

In our single‐year model of a *C. arvense* population in a sheep pasture in the absence of weed control operations, three compartments (Leathwick and Bourdôt 2012) are tracked over time *t* (months) starting on Sept 21st (mid‐spring) (*t *=* *0).

1. *r* ^*t:*^ Old root dry mass (g m^−2^). At Sept 21st (*t *=* *0), this comprises all underground overwintered plant material (thickened creeping root, adventitious buds, subterranean shoots, and feeding roots). This root mass formed over the previous year and has survived the winter. Over the subsequent 12 months (1 year) from Sept 21st–Sept 21st (*t *=* *0 to *t *=* *12), it decays. Any roots formed during this year join the “new root dry mass” compartment (see 3 below).
2. *n* ^*t:*^ Aerial shoot density (m^−2^). This comprises vegetative shoots (rosettes and bolting shoots) and reproductive shoots (budding, flowering, seeding).
3. *R* ^*t*^ *:* New root dry mass (g m^−2^). This is the root mass that is newly formed during the current year Sept 21st–Sept 21st (*t *=* *0 to *t *=* *12). As with old roots, it includes all subterranean tissues.

Model equations (eqs (1), (2), (3)) show the assumed positive inflows (recruitment) and negative outflows (mortality or loss) in each compartment:

(1) $$\text{Old}\;\text{root}\;\text{dry}\;\text{mass}:r^{t+1}=r^{t}-\mu_{r}^{t}r^{t}$$

(2) $$\text{Aerial}\;\text{shoot}\;\text{density}:n^{t+1}=n^{t}+c_{r}^{t}r^{t}+c_{R}^{t}R^{t}-\mu_{n}^{t}n^{t}$$

(3) $$\text{New}\;\text{root}\;\text{dry}\;\text{mass}:R^{t+1}=R^{t}+c_{B}^{t}b^{t}n^{t}-\mu_{R}^{t}R^{t}$$

where subscripts denote the compartment and superscripts time *t* (months). The time‐dependent mortality rate for each compartment is *μ* ^*t*^ month^−1^, and the recruitment rates of aerial shoots (number of aerial shoots per gram of root dry mass [old and new] per month) are $c_{r}^{t}$ and $c_{R}^{t}$, respectively. The “creeping root” parameter, $c_{B}^{t}$, is the transition rate from aerial shoots to the new root dry mass per month. The units of $c_{B}^{t}$ are “grams of new root dry mass per unit of aerial shoot dry mass per month”. The total average dry mass of *n* ^*t*^ aerial shoots at time *t* is *B* ^*t*^ **= *b* ^*t*^ *n* ^*t*^ where *b* ^*t*^ is the average dry mass (g) of one typical aerial shoot at time *t*.

**Figure 2:**
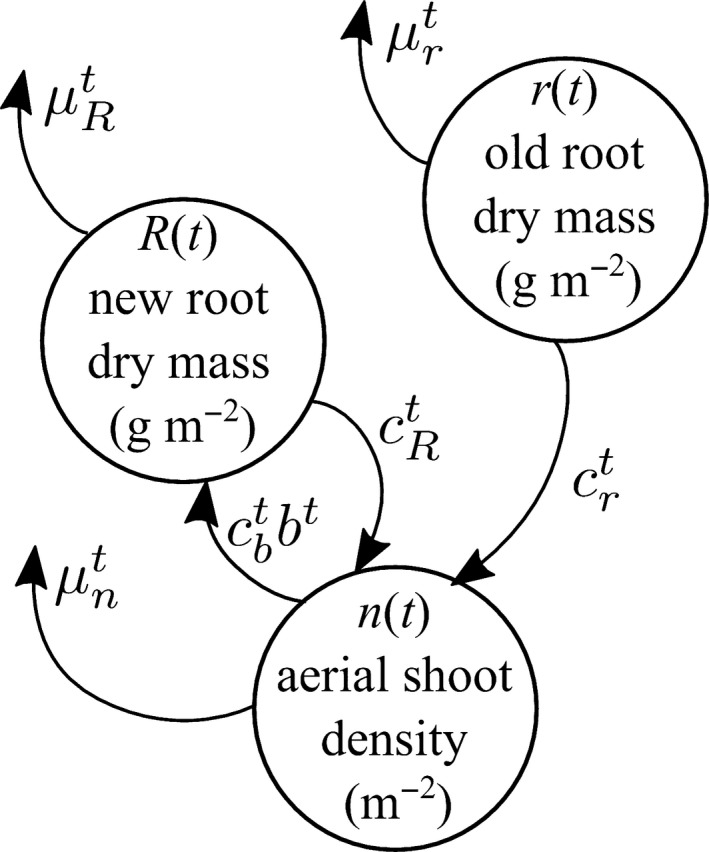
A schematic representation of the matrix model for the population dynamics of *Cirsium arvense* in a pasture illustrating its three components (old root, aerial shoots, and new root) and the transitions between them as defined in equations (1), (2), (3).

A schematic of the matrix model is given in Figure 2, and parameters are summarized in Table 1. Where possible, parameter values were estimated from experimental data collected in sheep pasture in New Zealand (Appendix S1).

**Table 1:** Input parameters for the *Cirsium arvense* population model

Parameter	Description	Value and/or unit
*t*	Time	Month
*t *= 0	Model start	Sept 21^st^
*r* ^*t*^	Old root dry mass	g m^−2^
*n* ^*t*^	Aerial shoot density	m^−2^
*R* ^*t*^	New root dry mass	g m^−2^
*r* ^0^	Initial old root dry mass (at *t *=* *0)	110.3 g m^−2^
*n* ^0^	Initial aerial shoot density (at *t *=* *0)	0 m^−2^
*R* ^0^	Initial new root dry mass (at *t *= 0)	0 g m^−2^
$\mu_{r}^{t}$	Loss rate in old root dry mass	0.19 month^−1^ (range 0.17–0.22)
$\mu_{n}^{t}$	Mortality rate of aerial shoots	0.20 month^−1^ *t *=* *0–4 (standard deviation 0.17, sample size 7)
		0.71 month^−1^ *t *=* *5–7 (standard deviation = 0.067, sample size = 3)
		1.0 month^−1^ *t *=* *8–11
$\mu_{R}^{t}$	Loss rate in new root dry mass	0 month^−1^ *t *=* *0–7
		0.13 month^−1^ *t *=* *8–11 (standard deviation 0.026, sample size = 3)
$c_{r}^{t}$ and $c_{R}^{t}$	Recruitment: number of aerial shoots m^−2^ per unit of root dry mass	0.16 g^−1^ month^−1^ *t *=* *0–7 (standard deviation 0.12, sample size = 7)
		0 g^−1^ month^−1^ *t *=* *8–11
$c_{B}^{t}$	Root dry mass production: root dry mass per unit of aerial shoot dry mass	0 month^−1^ *t *=* *0–2
		0.48 month^−1^ *t *=* *3–7 (standard deviation 0.047, sample size = 3)
		0 month^−1^ *t *=* *8–11
*h* ^*t*^	Average height of an aerial shoot	cm (see Fig. 2 and Appendix S1)
*b* ^*t*^	Average dry mass of an aerial shoot	g (see Appendix S1)

### Multiyear model

Despite 7.6% of old root mass surviving 12 months (Bourdôt et al. 2000), it is unlikely that this overwintered root mass present at the start of a growing season (September) could survive another winter (see Leathwick and Bourdôt (2012) p375 (Bourdôt et al. 2000)). Accordingly, for our multiyear model, we assume that at the beginning of each growing season (September), all surviving root mass is from the previous growing season. New roots and aerial shoots were assumed to be zero:

(4) $$\text{Old}\;\text{root}\;\text{dry}\;\text{mass}\;\left(g\;m^{-2}\right):r^{t+1}=R^{t}$$

(5) $$\text{Aerial}\;\text{shoot}\;\text{density}\;\left(m^{-2}\right):n^{t+1}=0$$

(6) $$\text{New}\;\text{roots}\;\text{dry}\;\text{mass}\;\left(g\;m^{-2}\right):R^{t+1}=0$$

where *t *+* *1 is Sept 21st.

The projection matrix **P** *,* which projects the population vector $X_{t}={\left[\begin{array}{c} r\\ n\\ R \end{array}\right]}_{t}$ from 1 year to the next (Sept 21st–Sept 21st), can be written:

(7) $$P=T\times A_{11}\times A_{10}\times \ldots \times A_{1}\times A_{0}$$

where

(8) $$A_{t}=\left[\begin{array}{ccc} 1-\mu_{r}^{t} & 0 & 0\\ c_{r}^{t} & 1-\mu_{n}^{t} & c_{R}^{t}\\ 0 & c_{b}^{t}b^{t} & 1-\mu_{R}^{t} \end{array}\right]$$

is the transition matrix from month *t* to month *t *+* *1 and

(9) $$T=\left[\begin{array}{ccc} 0 & 0 & 1\\ 0 & 0 & 0\\ 0 & 0 & 0 \end{array}\right]$$

is the transition matrix corresponding to equations (4), (5), (6). For example, **A**_0_ is the transition matrix from Sept 21st to Oct 21st, **A**_1_ is the transition matrix from Oct 21st to Nov 21st, etc.

Note that **A**_*t*_ stays constant for the same month over subsequent years and so the projection matrix **P** does not change from year to year and, for example, **X**_12_ = **P X**_0_ and **X**_24_ = **P X**_12_ etc. The dominant eigenvalue, *λ*, of the projection matrix **P** describes the long‐term dynamics of the population over time, with *λ *> 1, 1, and <1 corresponding to long‐term population increase, stasis, and decrease, respectively (Caswell 2001).

### Multiyear model incorporating mowing

**Figure 3:**
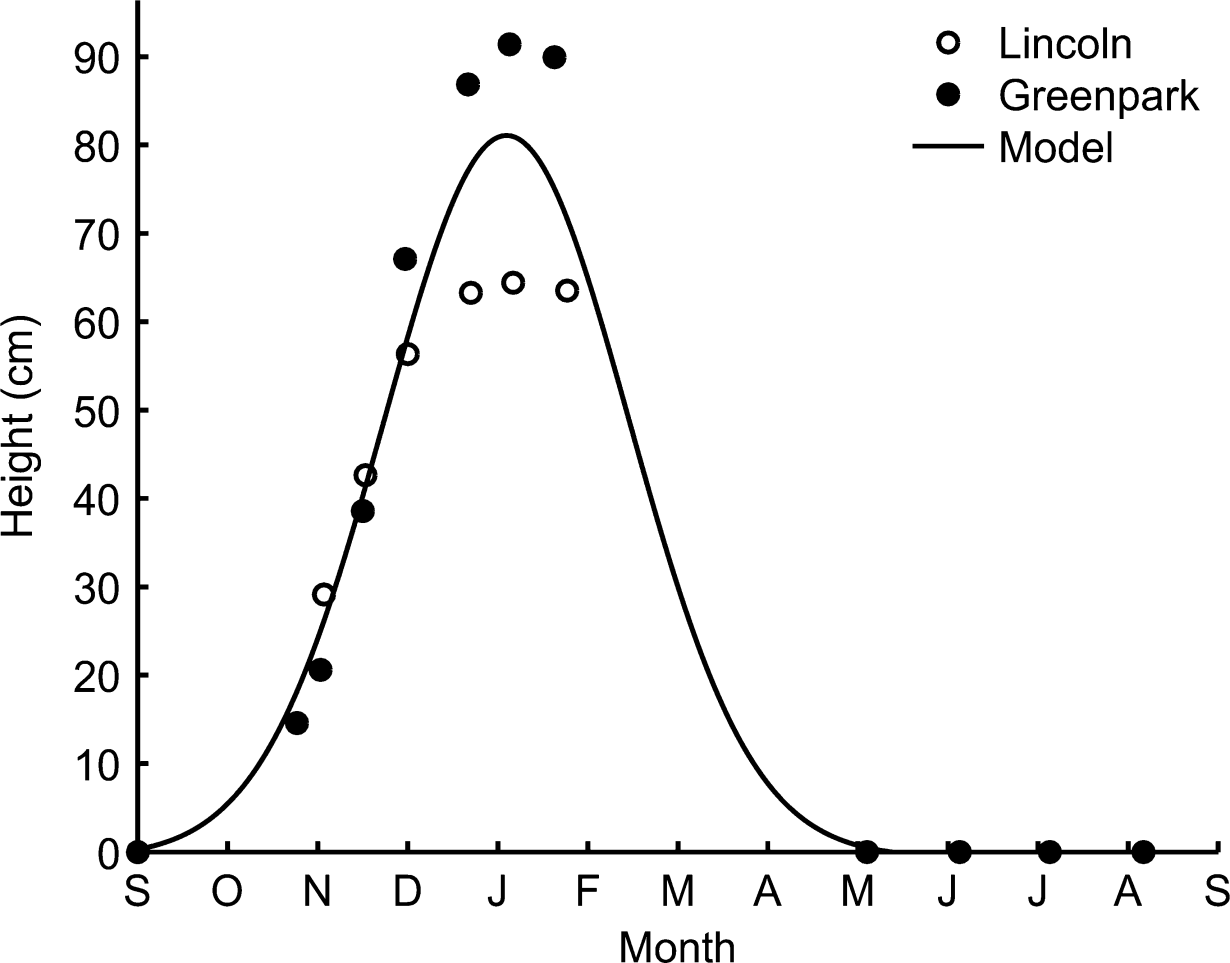
Data for the height of the aerial shoots of *Cirsium arvense* (circles) as measured in different months of the year in two populations of the weed in pasture in New Zealand in the absence of control operations (fig. 3A and B in Cripps et al. 2011). See Appendix S1 and the corresponding least‐squares fitted curve (solid line).

Mowing was incorporated into the model via the shoot height curve (see Fig. 3 and Appendix S1) which was modified so that aerial shoot heights, from the day of mowing, were calculated as if from time *t *=* *0. We firstly assumed that this regrowth was independent of the month that mowing took place. We then considered the possibility of autumnal root bud dormancy (Andersson et al. 2013), where auxiliary buds on the shoots below mowing height do not resprout after mowing. Autumnal root bud dormancy was incorporated into the model by setting the transition rate from roots to aerial shoots to zero after autumnal mowing.

The model assumes that utilization of root reserves by regrowing shoots does not occur. This may be reasonable as postcutting regrowth in otherwise intact plants occurred almost solely from auxiliary buds on the cut shoot stumps in the Swedish study (Andersson et al. 2013), rather than from deeper‐positioned root buds which consume root reserves during their growth to the soil surface (Zhang et al. 2011).

A number of mowing regimes were simulated. First, for model validation, combinations of early (Nov 21st), mid (Jan 21st) and late (Feb 21st) season mowing events were considered. For each combination, model outputs “autumnal root biomass” and “aerial shoot biomass duration” were compared with experimental data from Bourdôt et al. (1998). Here, we define the autumnal root biomass as the total root dry mass (g m^−2^) as at May 21st (*t *=* *8) (i.e., *r* ^*t *= 8^ + *R* ^*t *= 8^) which was the closest date (in the model) to the experimental sampling (first week of June). The model's estimate of the aerial shoot biomass duration (g days) was defined, in accordance with the experimental data, as the area (using the trapezoidal rule of integration) under the total aerial biomass versus time curve (converted to days i.e., 30 × *n* ^*t*^ **× *b* ^*t*^) between *t *=* *2 (Nov 21st) and *t *=* *7 (Apr 21st).

Secondly, to consider more effective mowing, the mowing regimes “mow once a year” (7 possibilities) and “mow twice a year” (21 possibilities) were considered. It was assumed that mowing could take place on the 21st of any month from October to April inclusive. September was excluded because *C. arvense* shoots are below mower height in early spring. Similarly, May–August were excluded as *C. arvense* dies off over these late autumn/winter months. The mowing combinations were given identification acronyms according to the first letter of the mowing month that is mowing on January 21st and March 21st was encoded “JM” etc.

#### Elasticity analysis

The elasticity matrix, **E**_*t*_ for each monthly transition matrix **A**_*t*_, was calculated using methods described in Lesnoff et al. (2003), and Caswell and Trevisan (1994). Elements of this matrix represent how a small perturbation in the corresponding element of the monthly transition matrix, **A**_*t*_ (eq. 8), affects the long‐term population growth rate *λ*. The column sum of the elasticity matrix gives the contribution of each compartment to *λ* in that month.

## Results

### One‐year model

#### Model output using default parameters

**Figure 4:**
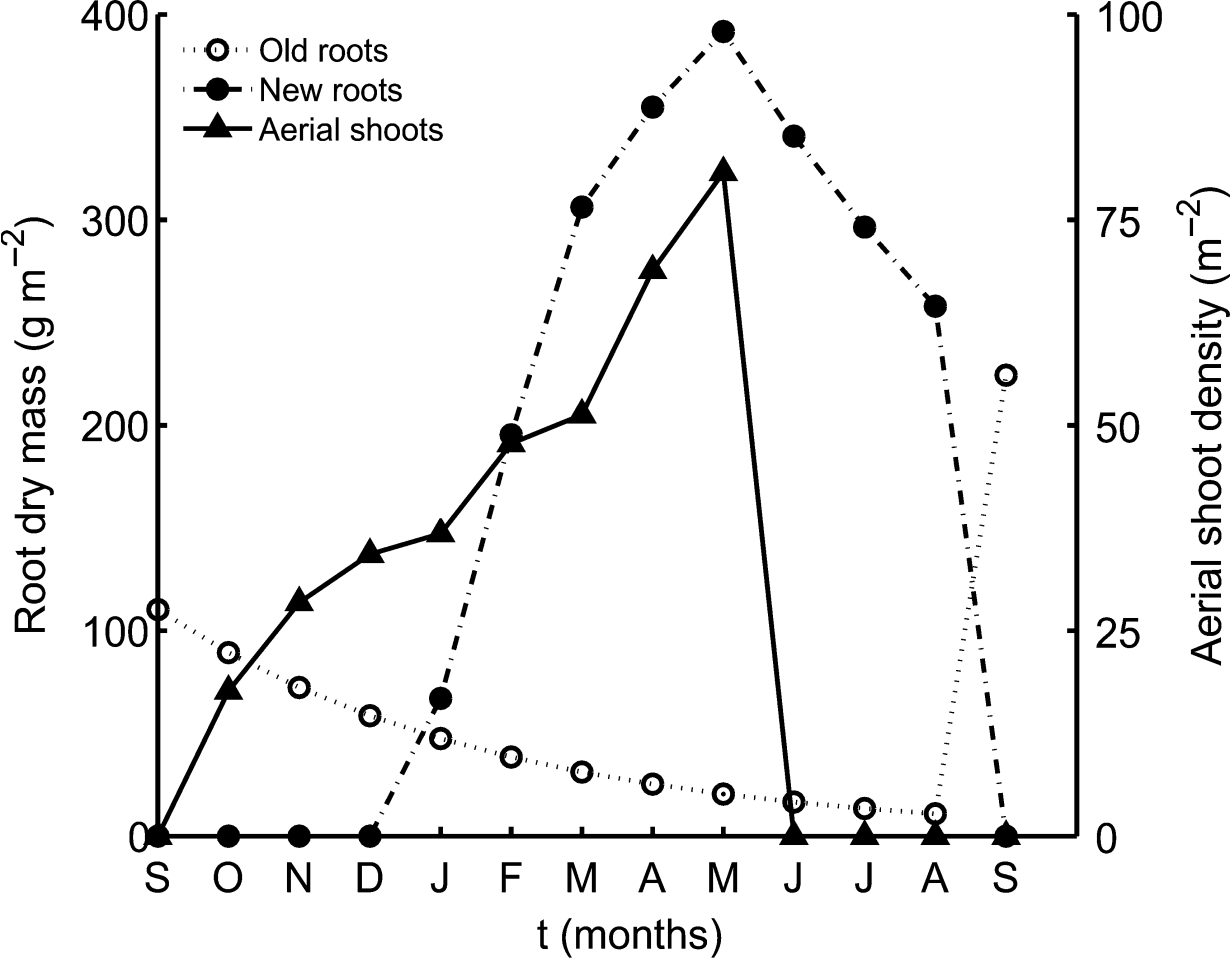
Trajectories for a one‐year period (Sept 21st [*t *=* *0] → Sept 21st [*t *=* *1]) for each of three compartments (old roots, aerial shoots, and new roots) generated by the *Cirsium arvense* population model (equations (1), (2), (3)) in the absence of mowing with parameter values as given in Table 1.

In the first year, with default parameter inputs, the model population density of *C. arvense* shoots followed a trajectory over time as depicted in Figure 4. Old roots decayed from an initial mass of 110.3 g m^−2^ at a constant rate throughout the year. Aerial shoot density increased steadily from zero m^−2^ in September through to around 50 m^−2^ in March, this was followed by a more rapid increase in density from March to May (~80 m^−2^) and then a subsequent rapid winter decline. New root dry mass began to form from December and increased steadily until the end of May (~400 g m^−2^), subsequently senescing over the winter months. What was left of the new root mass at the end of the year became the old root mass.

The eigenvalue of the projection matrix **P** for default model parameters was *λ *= 2.03, implying that, in the long term, shoot population density doubles each year.

#### Model validation

The trajectory for aerial shoot density is similar in shape to that calculated for percentage ground cover in Bourdôt et al. (2016). Both the aerial shoot and creeping root trajectories are realistic, corresponding to values that have been observed on medium to highly infested sheep and beef farms (Cripps et al. 2010). In addition, the rapid increase in aerial shoot density over the autumn in the model (“autumn flush”) is a phenomenon observed in the field (Berner et al. 2013; Cripps et al. 2014).

**Figure 5:**
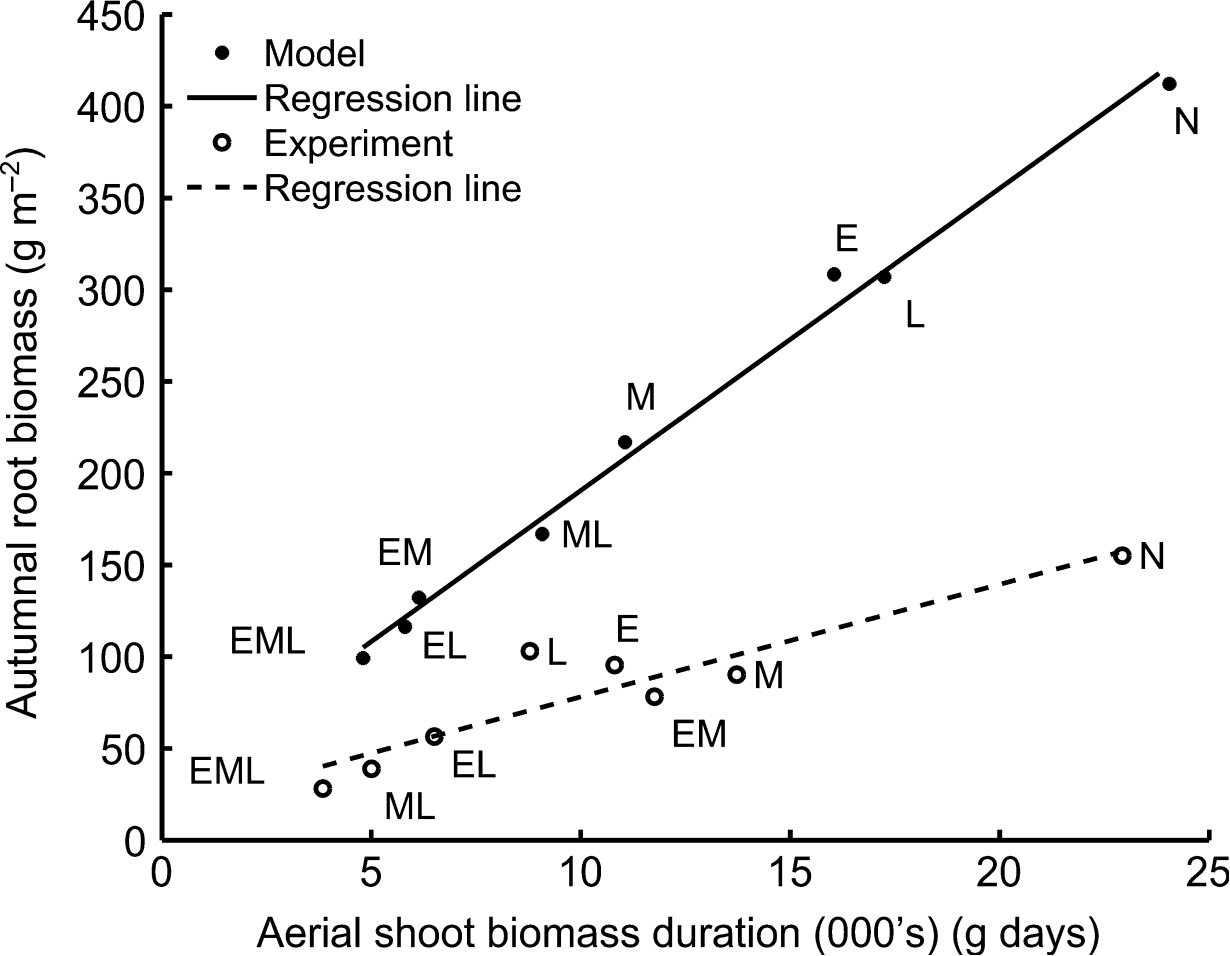
The relationship between autumnal root biomass, ARB (g m^−2^), and aerial shoot biomass duration SBD (g days) in *Cirsium arvense* as determined by an experiment which manipulated time of year and frequency of mowing (Bourdôt et al. 1998) [refitted regression ARB = 13.83 + 0.0064 × SBD (‐‐‐‐) without the zero‐intercept constraint] and by model simulation [Fitted regression ARB = 25.87 + 0.016 × SBD (^____^)]. Time of year when mown shown as N “not mown”, E “early season”, “M” mid‐season, and “L” late season where E = November (spring), M = January (summer), and L = February/March (late summer/early autumn).

The relationship between the autumnal root biomass and aerial shoot biomass duration was found to be linear in the field experiments by Bourdôt et al. (1998), and our model confirmed this linear relationship (Fig. 5).

### Multiyear model

#### Simulating mowing

**Figure 6:**
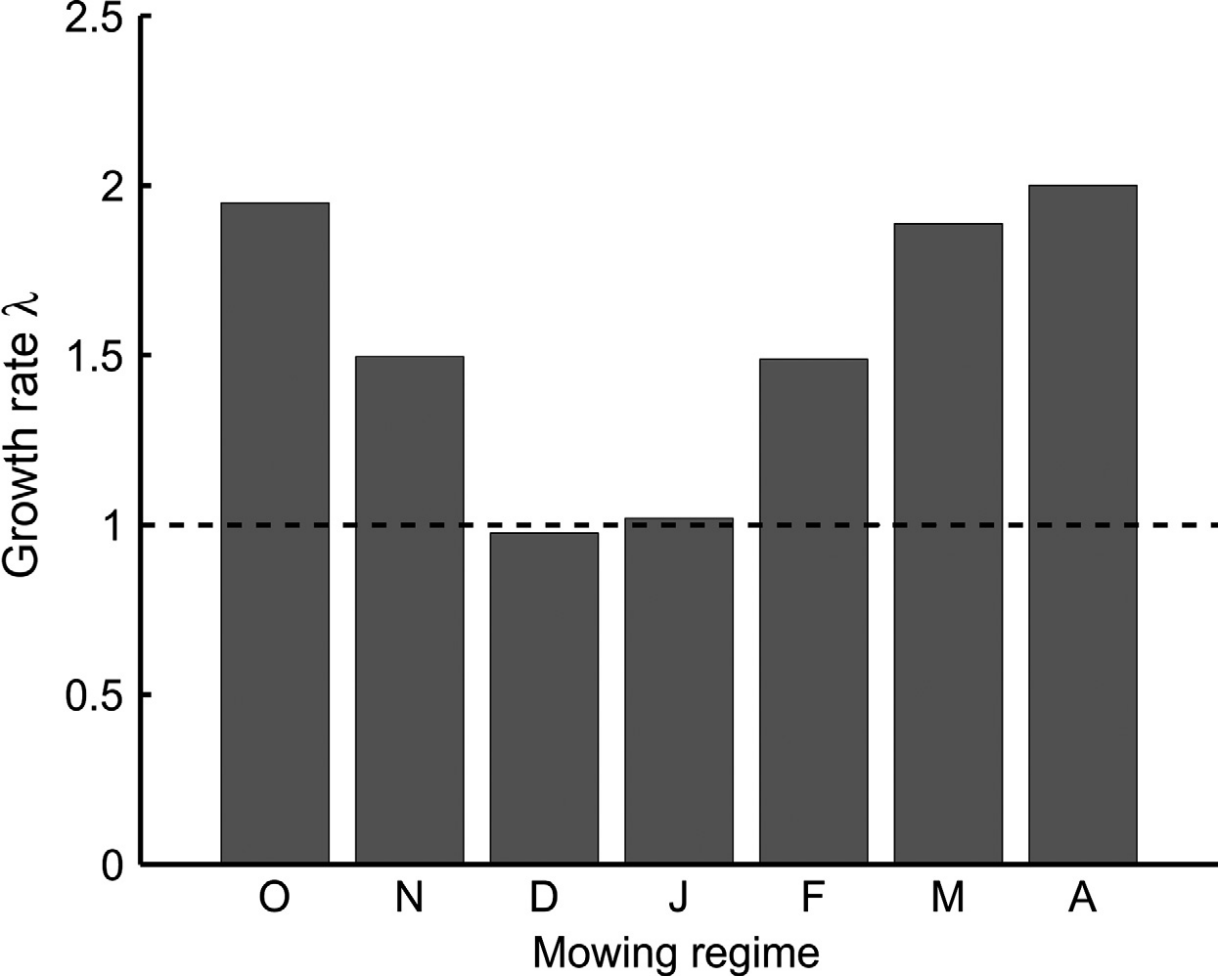
Population growth rate *λ* in *Cirsium arvense* for different modeled mowing regimes involving one mowing per year (spring [O, N]; summer [D, J, F]; autumn [M, A]).

When the simulated mowing regime was “mow once a year”, the model produced smaller values for autumnal root biomass (Jan 21st 217 g m^−2^) and for aerial shoot biomass duration (Jan 21st 11,058 g days) when the mowing event was imposed in mid‐summer (January) as compared to both earlier (spring) of later (autumn) in the growing season (Fig. 5). The population growth rates were also smallest for the simulated mid‐summer mowing regimes and were close to unity for December (*λ *= 0.98) and January (*λ *= 1.02) (Fig. 6).

**Figure 7:**
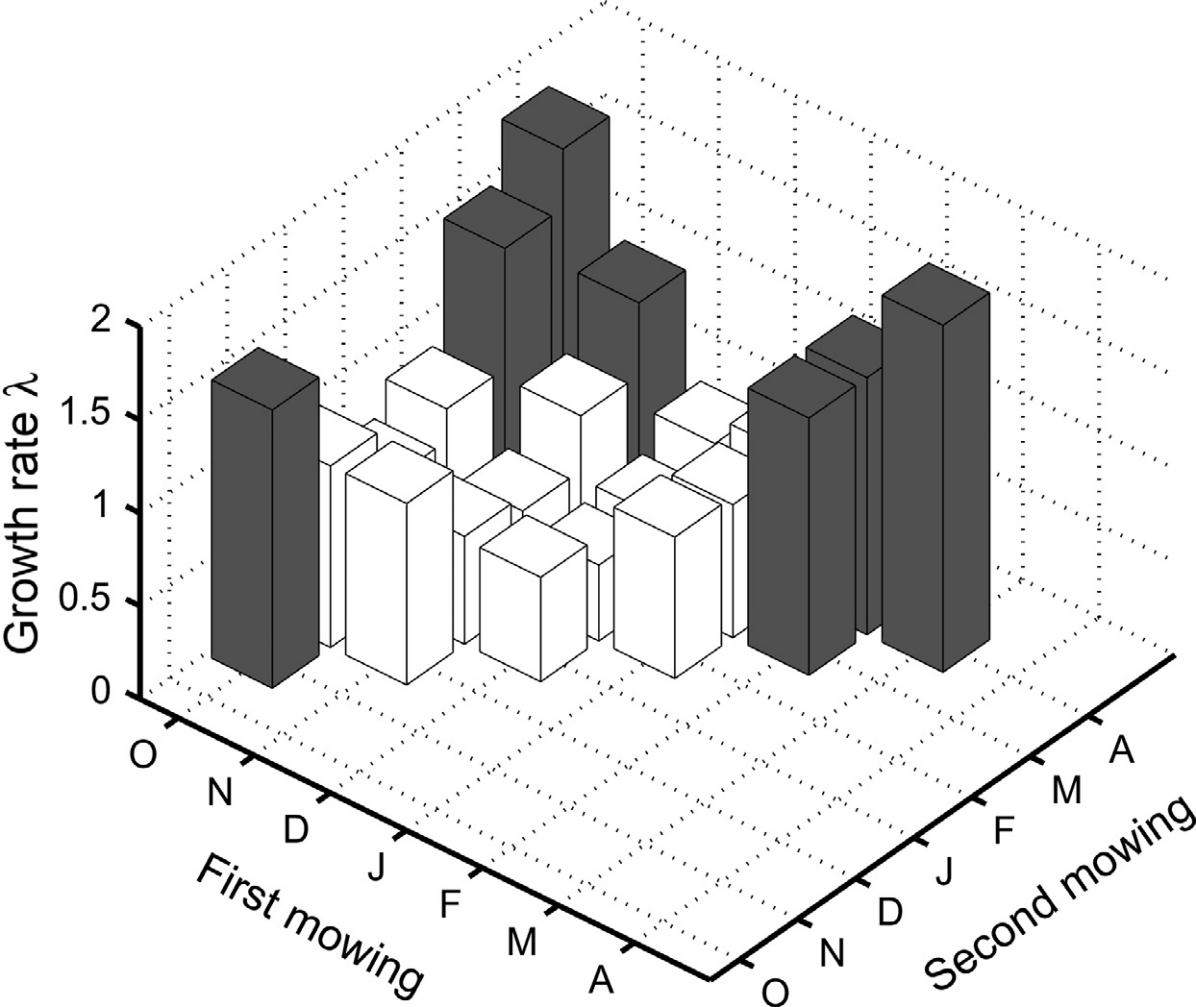
Population growth rate *λ* (white when *λ *< 1 and gray otherwise) in *Cirsium arvense* for different modeled mowing regimes involving two mowings per year (spring [O, N]; summer [D, J, F]; autumn [M, A]).

When mowing twice a year, the optimal regime was the combination of December and February mowing (*λ *= 0.41, autumnal root biomass = 99 g m^−2^, aerial shoot biomass duration = 5,133 g days) (Fig. 7). Seven of the 21 possible combinations for mowing twice a year resulted in an eigenvalue greater than unity (ON, OM, OA, NA, FM, FA, MA). All other combinations corresponded to long‐term population decreases. The mowing regimes NF, DF, and DM had eigenvalues less than or equal to 0.5, that is long‐term population density at least halved each year.

Incorporating autumnal root bud dormancy reduced *λ* by an average of approximately 8% in all one and two‐yearly mowing regimes that included the month of March. This reduction did not cause any of the *λ* values to fall below unity. Autumnal root dormancy did not affect *λ* values for April mowing.

#### Elasticity

**Figure 8:**
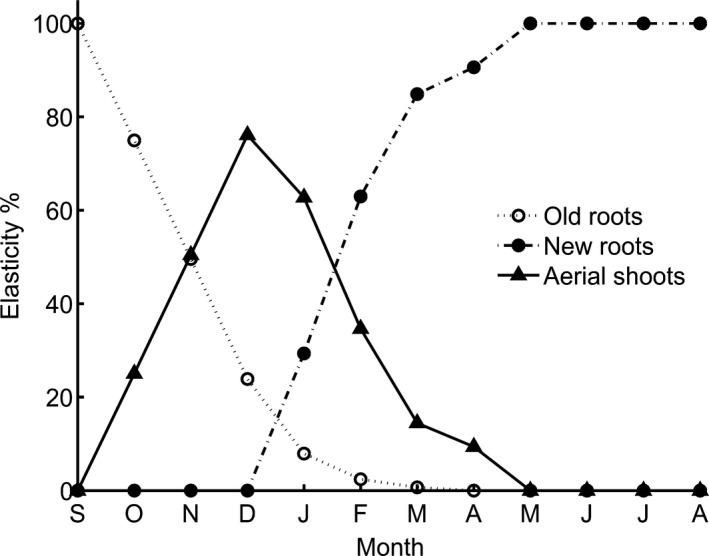
The elasticity (%) is the sum of the columns of the elasticity matrix, **E**_*t*_ for each monthly transition matrix **A**_*t*_ and gives the contribution of each compartment to the population growth rate *λ* in *Cirsium arvense* in that month (spring [S, O, N]; summer [D, J, F]; autumn [M, A, M]; winter [J, J, A]).

In spring (Sept–Nov), the biggest contribution to population growth (*λ*) came from the overwintered root compartment through old root survival and recruitment of new aerial shoots from the overwintered root mass (Fig. 8). In early and mid‐summer (Dec and Jan), aerial shoot transitions (shoot survival and new root dry mass) via the photosynthetic opportunity afforded by the aerial shoot population were the most important contributors to *λ*. The new root dry mass compartment had the highest contribution to *λ* from February to August.

## Discussion

Model input parameters were chosen to be seasonal, so output population trajectories also showed seasonal behaviors as expected (Fig. 4). In the absence of mowing, the autumn flush of new aerial shoots occurred because inputs in this compartment (new thistle rosettes arising from the creeping roots) were greater than outputs (aerial shoot mortality) during this time. The long‐term population growth rate *λ *= 2.03 implied that the population density eventually doubled every year in the absence of mowing. That this may be an overestimate is supported by the modeled relationship between autumnal root biomass and aerial shoot biomass duration lying above the relationship derived experimentally (Fig. 5). By comparison, Chalak et al. (2008) estimated a maximum population annual growth rate of 2.5 (with a range of 2.3–2.7). Unfortunately, data to validate these population growth rate estimates (in the absence of mowing) are lacking as most experiments follow shoot population density within only a single growing season. An exception to this is Bourdôt et al. (2006) where 4 years of observations in a sheep pasture indicated that shoot population density remained more or less stable (*λ *≈ 1). Also Donald (1993) estimated that shoot densities in late summer were approximately 40% of those the previous year (*λ *= 0.4). However, both of these experimental estimates of *λ* were derived from shoot density data collected within patches of *C. arvense*. Because creeping roots move off plots over time, the shoot density data collected most likely underestimate the actual changes in shoot population size and density that were occurring at the larger scale.

In the model, the rate of increase in the new root dry mass peaked throughout January and February (Fig. 4) and this was due to the peak in photosynthetic opportunity provided by the aerial shoots which peaked in size in the preceding months (December and January). December and January would therefore appear to be the optimal months for a once‐per‐year annual mowing regime (Figs. 5, 6), and this is supported by the elasticity analysis which showed that the aerial shoots have their greatest contribution to the long‐term population growth rate (*λ*) at this time of year (Fig. 8). However, optimal annual once‐per‐year mowing apparently results in insufficient reduction in the whole season photosynthetic opportunity to give a long‐term population growth rate, *λ*, of less than 1.0 and hence population decline (Fig. 6). Twice‐yearly mowing is required to achieve that (Fig. 7) but not all twice‐yearly mowing combinations were sufficient, emphasizing the importance of the timing of mowing in the field (Zhang and Shea 2012).

#### Future work: Stochastic parameters

By comparing theoretical management strategies, the model goes beyond the existing empirical data and provides guidance for the manager on how to optimally position two mowing events within a growing season so as to achieve a long‐term declining population of *C. arvense*. This assumes twice‐yearly mowing takes place every year into the foreseeable future. We have not considered how many years it would take to reach extinction. Ideally, this would involve stochastic simulations and would require sampling parameters from distributions currently unknown. Further analyses, beyond the scope and resources of the current project, would include defining such parameter distributions.

#### Future work: Biological controls

In an earlier *C. arvense* population modeling study, biological control agents were incorporated into a Leslie matrix model by Forsyth (1985). Simulations indicated that a single agent could not alone reduce population size and that a combination of agents is required. In the future, we intend to generalize our model to extend and further validate the research of Forsyth by evaluating the effects of a range of contemporary biological controls (and mowing) applied both individually and in combination (possibly with synergistic effects). For example, the rust fungus, *Puccinia punctiformis* (naturally present at low frequency in most *C. arvense* populations (Cripps et al. 2009)), resides in the overwintering *C. arvense* root system and kills young emerging shoots resulting in population declines (Berner et al. 2013). In New Zealand, autumn inoculation of *C. arvense* rosettes with the teliospores of the fungus resulted in a higher incidence of systemic disease compared to ambient disease in control plots (Cripps et al. 2014). In the Northern Hemisphere, Demers et al. (2006) showed that over time, September (early autumn) mowing, as compared to no mowing, increased the proportion of *C. arvense* shoots infected by the rust. The plant's response to inoculation could be incorporated into the model by reducing the spring transition rate from old root dry mass to aerial shoot density. Many combinations of mowing and inoculation strength could then be considered with the goal of elucidating an optimal combination.

Similarly, the biocontrol agent, *Cassida rubiginosa*, defoliates *C. arvense* throughout most of the growing season, particularly from late spring to mid‐summer (November to January). Adults and larvae of the beetle feed on the leaf mesophyll tissue, stripping the plant of its photosynthetic capacity thus restricting the plants ability to produce new overwintering root mass. In a *C. arvense* population in New Zealand, the beetle was observed to occasionally kill aerial shoots, and on average caused 63% defoliation (Cripps 2013). An extension of the current model might be able to specify the amount of defoliation required by biocontrol agents to effectively reduce the long‐term population growth rate of the weed. The plant's response to defoliation due to beetle feeding could be incorporated into the model by reducing the transition from aerial shoot density to new root dry mass.

## Conclusion

From the population model that we have developed for *C. arvense* in permanent grassland, where the size of an ephemeral overwintering root bud population is a linear function of the photosynthetic opportunity attributable to the aerial shoots during the preceding growing season, two conclusions may be drawn concerning the influence of mowing. First, an annually repeated single mowing, regardless of the time when this is imposed on the shoots during each growing season, is unlikely to cause long‐term population decline. Second, mowing can be effective in reducing populations of *C. arvense* in pasture in the long term if conducted twice each year. According to the model, the optimal combination is when the initial mowing is conducted in mid spring followed by a subsequent mowing from mid summer to early autumn. These mowing regimes reduce the photosynthetic opportunity of the *C. arvense* population and hence its ability to form the overwintering creeping roots upon which population growth depends.

Over a typical growing season, *C. arvense* populations in native and introduced regions have similar dynamics (Cripps et al. 2010), suggesting that the model presented here can be parameterized and applied to pasture in other parts of the temperate world where *C. arvense* is also a problematic weed (Tiley 2010; Guggisberg et al. 2012).

## Conflict of Interest

None declared.

## Supporting information


**Appendix S1.** Parameter value estimation for the matrix model.
