# Supplementary material for: Mowing strategies for controlling Cirsium arvense in a permanent pasture in New Zealand compared using a matrix model
Source: Ecol Evol. 2016 Mar 28;6(9):2968–77. doi: 10.1002/ece3.2090 (PMC4809806; doi:10.1002/ece3.2090)
Supplement: Supplementary file 1 — Appendix S1. Parameter value estimation for the matrix model. [file ECE3-6-2968-s001.docx]

# Supporting Information for:

# Mowing strategies for controlling *Cirsium arvense* in a permanent pasture in New Zealand compared using a matrix model

Graeme Bourdôt, Britta Basse, Michael Cripps

AgResearch Limited, Private Bag 4749, Christchurch 8140, New Zealand

[graeme.bourdot@agresearch.co.nz](mailto:graeme.bourdot@agresearch.co.nz), [britta.basse@cpit.ac.nz](mailto:britta.basse@cpit.ac.nz), [mike.cripps@agresearch.co.nz](mailto:mike.cripps@agresearch.co.nz)

*Correspondence:* Dr Britta Basse,

Department of Humanities

Education and Applied Research Division

Te Waka Ako, Te Hoe Aronui

CPIT Aoraki

PO Box 540, Christchurch 8140

New Zealand

[britta.basse@cpit.ac.nz](mailto:britta.basse@cpit.ac.nz)

+64 3 940 8218

**Parameter value estimation for the matrix model**

The methodology for obtaining the parameter values used in the *C. arvense* matrix model is described in detail here. Where possible, these values were estimated from experimental data collected in sheep pasture in New Zealand.

*Initial conditions*

In New Zealand, all aerial shoots of *C. arvense* senesce in the late autumn (May) but the root system persists through the winter (Bourdôt *et al.* 1995). Adventitious buds on the over-wintered roots develop into subterranean shoots that emerge through the soil causing a “flush” of aerial shoots (vegetative leafy rosettes) in mid spring (late September – early October) (Leathwick & Bourdôt 2012) .

We assumed a starting date in the model of Sept 21^st^ (*t* = 0) just prior to the spring flush of aerial shoots. The initial density of aerial shoots m^-2^ is *n*^0^ = 0 m^-2^ and new roots have yet to begin forming (*R*^0^ = 0 g m^-2^). The initial over-wintered root dry mass, *r*^0^ = 110.3 g m^-2^, was estimated from 24 soil core (100 mm diameter) samples (300 mm deep) taken from a sheep-grazed pasture at the AgResearch experimental farm at Templeton, New Zealand on Sept 23^rd^ 1992 (1103 kg per ha from Bourdôt *et al.* (1995)).

*Average biomass and height of an aerial shoot*

In an experiment in mid-summer in Switzerland (Cripps *et al.* 2010) the height (cm) of an aerial shoot was a good predictor of its dry mass (g). The relationship (M. Cripps, unpublished data) is

| $\text{ }b^{t}= 0.9094\times\exp(0.0257\times h^{t})$ | eqn 1 |
| --- | --- |

where $b^{t}$and $h^{t}$are the respective average biomass (g) and average height (cm) of a typical aerial shoot in month *t* (R^2^ = 0.8775). We assumed this relationship applies in New Zealand pastures throughout the year and that the biomass is zero when the height is zero.

Shoot height $h^{t}$(cm) (averaged over two experimental sites) increased during spring and flattened out in summer (control data in Figs. 3 A & B in Cripps *et al.* (2011)) in New Zealand (Fig. 1). We assumed that during autumn shoot heights decline, reaching zero by the end of autumn when all aerial shoots have senesced. The data was optimally (least squares) fitted to the Gaussian function described in equation 2 using the *Octave* function *nonlin_curvefit* (Eaton, Bateman & Hauberg 2009) giving *μ* = 4.10 (the position parameter), *σ* = 1.36 (the standard deviation), *c*_1_ = 81.64 (the height) and *c*_2_ = −0.57 (the vertical shift parameter).

| $\hat{h}^{t}=c_{1}\exp\left(\frac{-\text{(}t-\mu\text{)}^{2}}{2\sigma^{2}}\right)+ c_{2}.$ | eqn 2 |
| --- | --- |

We assumed that this height curve (and hence the biomass relationship equation 1) holds for all shoot densities based on the findings of Leathwick *et al.* (2006) where the mass of the average shoot was unaffected by plant density. Heights were truncated to zero from May 21^st^ (*t* = 8) onwards (winter) to simulate the winter deciduous phase of the species.

*Loss of old root dry mass*

Inhibiting new root growth in a *C. arvense* population by mowing to ground level weekly, Bourdôt, Leathwick and Hurrell (2000) found that only 7.6% (± 2.5% (95% confidence)) of the root mass present in May (late autumn) survived 12 months. The constant presence of decayed roots implied that decay of old (over-wintered) roots is a continuous process. For the model, a constant rate of decline ($\mu_{r}^{t}=\mu_{r}$per month) in the old root dry mass (g m^-2^) was assumed, $\mu_{r}$being calculated so that the old root mass was reduced to 7.6% over the 12 month period from Sept 21^st^ (*t* = 0) to Sept 21^st^ (*t* = 12). From the model (equation 1 in main document),$r^{12}=(1-\mu_{r}^{t})^{12}r^{0}$and month^-1^ there is a loss of 19% of old root dry mass (g m^-2^) per month. The 95% confidence interval limits for experimental root dry mass persistence (5.1 – 10.1%) correspond to a mortality rate range of 0.17 – 0.22 month^-1^.

*Mortality* *of* *aerial shoots*

The spring to late summer loss rate of aerial shoots, $\mu_{n}^{t}$ month^-1^, where *t* = 0 – 4 (Sept 21^st^ – Jan 21^st^) was estimated using the data from non-treated plots in the bio-herbicide efficacy experiment described in Bourdôt *et al.* (1995) where the aerial shoots and their deaths were counted on nine occasions from Oct 13^th^ to Mar 7^th^ 1992. Cumulated deaths approximated linearity over time and the shoot population remained approximately constant. From this data, a per capita monthly shoot death rate was calculated and averaged over the spring to late summer censuses (Oct 13^th^ – Jan 23^rd^) giving $\mu_{n}^{t}$ = 0.20 month^-1^ (standard deviation 0.17, sample size 7).

Autumnal aerial shoot mortality $\mu_{n}^{t}$ month^-1^, where *t* = 5 – 7 (Feb 21^st^ – Apr 21^st^) was estimated by considering the experimental data from only reproductive shoots, a cohort which has no inputs in autumn, hence the solution of model equation (equation 2 in main document) is$n^{t}=(1-\mu_{n})^{t}n^{0}$ where *t* = 0 is the first autumnal census and *n*^0^ is the number of reproductive shoots at this time. This solution curve was fitted using the Octave software function *leasqr* (Eaton, Bateman & Hauberg 2009) to annual autumnal declines (1993 – 1995) in reproductive shoots using non-treated census data from Bourdôt *et al.* (2006), giving an average mortality rate of $\mu_{n}^{t}$ = 0.71 month^-1^ (standard deviation = 0.067, sample size = 3). This mortality rate was assumed to apply to all aerial shoots.

By late May (late autumn) there is no living tissue above the ground (Leathwick & Bourdôt 2012) therefore we assume that winter-time mortality is 100% per month i.e. $\mu_{n}^{t}$ = 1 month^-1^ where *t* = 8 – 11 (May 21^st^ – Aug 21^st^).

*Loss of new root dry mass*

The loss of new root dry mass,$\mu_{R}^{t}$ month^-1^ (g m^-2^), was assumed to be zero over spring, summer and autumn during when these new roots are growing.

The winter-time loss of new root dry mass (g m^-2^ month^-1^ ) was estimated using data from non-treated plots in the experiment described in Bourdôt *et al.* (2006) where the root wet mass (g m^-2^) was measured approximately monthly from Oct 29^th^ 1992 to Sept 6^th^ 1996. From Nov 17^th^ 1993 until Aug 2^nd^ 1995, the dry mass (g m^-2^) was also measured. A linear regression analysis of these data indicated that root dry mass (g m^-2^) is approximately 20% of root wet mass (g m^-2^, R^2^ = 0.78). Measurements of root wet mass (g m^-2^) in the winter months of the first year (1993 on Jun 30^th^, Jul 21^st^, Aug 4^th^, Aug 25^th^, Sept 16^th^) were multiplied by 20% to get the dry mass (g m^-2^) then the loss rate per month was calculated. From this an average mortality rate was calculated as $\mu_{R}^{t}$ = 0.13 month^-1^ in June, July and August (standard deviation 0.0254, sample size = 3, *t* = 8 – 11 or May 21^st^ – Aug 21^st^).

*Recruitment of aerial shoots from old roots*

The value of $c_{r}^{t}$ month^-1^, the number of new shoots per gram of old root dry mass, was estimated from untreated census data in the bio-herbicide experiment described in Bourdôt *et al.* (1995) where new shoots (“births”) were counted over time. For our estimate, only census dates up to midsummer were considered based on the assumption that new shoots (births) during this period would be mainly from the old (over wintered) root system.

New shoots present at each census were converted to births m^-2^ month^-1^ and then to “births g^-1^ of old root dry mass” by estimating the mass of old roots at each census (Table 1). An initial (*t* = 0, Sept 21^st^) estimate of old root dry mass of 76.8 g m^-2^ was calculated by noting that on Oct 13^th^ 1991 there were on average 43 aerial shoots m^-2^ in the untreated plots, and assuming the relationship:

$$\left(\begin{matrix} \text{Initial root dry mass} \\ \text{(g m}^{\text{-2}}\text{)} \end{matrix}\right)\times\left(\begin{matrix} \text{Root buds } \\ \text{(g}^{\text{-1}}\text{ root dry mass)} \end{matrix}\right)\times\left(\begin{matrix} \text{proportion that elongate to} \\ \text{become subterranean shoots} \end{matrix}\right)=43.$$

[Bourdôt *et al.* (2006](#_ENREF_2)) state “there are on average 2.8 root buds per gram of root dry mass and approximately 20 % of these elongate to form subterranean shoots”. If we assume that all of these become aerial shoots then on Oct 13^th^ 1991 when 43 aerial shoots were counted:

$$\left(\begin{matrix} \text{Root dry mass} \\ \text{(g m}^{\text{-2}}\text{)} \end{matrix}\right)=\frac{43}{2.8\times0.2}=76.8 \text{g.}$$

Old root dry mass estimates for later censuses (after Oct 13^th^ 1991) were calculated according to exponential decay of 19% month^-1^.

The transition rate of aerial shoots from root dry mass,$c_{r}^{t}$, was estimated as the average number of births g^-1^ root dry mass month^-1^ for all census dates until midsummer (January) giving $c_{r}^{t}$ = 0.16 g^-1^ month^-1^ (standard deviation = 0.12, sample size = 7). After midsummer, plant resources are directed to new root growth, so we assumed that shoot births were no longer predominantly from the old root compartment. The transition rate was assumed constant for these remaining non-winter months i.e. $c_{r}^{t}$ = 0.16 g^-1^ month^-1^,  *t* = 0 – 7 (Sept 21^st^ – Apr 21^st^).

For the winter months we assume $c_{r}^{t}$ = 0 (*t* = 8 – 11 May 21^st^ – Aug 21^st^) since field observations indicate that shoots remain below ground during this time (Leathwick & Bourdôt 2012).

*Recruitment of aerial shoots from new roots*

The transition rate to aerial shoots was assumed equal for old and new roots, i.e. $c_{R}^{t}$= $c_{r}^{t}$. This seems reasonable but cannot be verified since old roots are distinguishable from new roots and their attendant aerial shoots in the field.

*Transition from aerial shoots to new root dry mass*

The formation of new roots begins a few weeks after the new shoots emerge from the ground but their growth at this time is limited because priority is given to the growth of the newly emerged adventitious shoots (Leathwick & Bourdôt 2012). We assumed that the increase in new root dry mass (g m^-2^) is negligible in spring while aerial shoots comprise vegetative shoots, i.e. $c_{B}^{t}$ = 0 when *t* = 0, 1, 2, (Sept 21^st^ – Nov 21^st^).

Since there is no living tissue above the ground during the winter (Leathwick & Bourdôt 2012) we assumed that there is no growth of the roots i.e. $c_{B}^{t}$ = 0 per month *t* = 8 - 11 (May 21^st^ – Aug 21^st^).

To calculate the increase in subterranean root dry mass (g m^-2^ month^-1^) during summer and autumn, we used data from a neighbour height experiment (Bourdôt *et al.* 2015) where *C. arvense* plants were established using root fragments in 80 crates (subplots) (4 treatments x 4 replicates x 5 harvests). The treatments were 4 trimming scenarios where neighbouring pastures plants were kept trimmed to a certain height. Here we use the data from the “short pasture” trimming treatment which simulated a sheep grazed pasture. The root dry mass increased linearly (approximately 7.8 g month^-1^) over the first three months (Total root dry mass = 7.8 *t* + 1.4, R^2^ = 0.99). The total aerial shoot dry mass also increased linearly (about 1.6 g m^-2^ month^-1^ or *B^t^* = 1.6 *t* + 13.1, R^2^ = 0.86). So $c_{B}^{t}$ is the increase in the dry mass of creeping root per aerial shoot dry mass per month i.e. $c_{B}^{t}$ = 7.8 grams/*B^t^* per month. If *t* is considered only over the range of the data and averages used to estimate $c_{B}^{t}$ , then $c_{B}^{t}$ = 0.48 (*n* = 3, standard deviation = 0.0469, 95% CI 0.47 to 0.49).

# References

Bourdôt, G.W., Harvey, I.C., Hurrell, G.A., & Saville, D.J. (1995) Demographic and biomass production consequences of inundative treatment of *Cirsium arvense* with *Sclerotinia sclerotiorum*. *Biocontrol Science and Technology*, **5**, 11-25.

Bourdôt, G.W., Hurrell, G.A., Saville, D.J., & Leathwick, D.M. (2006) Impacts of applied *Sclerotinia sclerotiorum* on the dynamics of a *Cirsium arvense* population. *Weed Research*, **46**, 61-72.

Bourdôt, G.W., Leathwick, D.M., & Hurrell, G.A. (2000) Longevity of Californian thistle roots. *New Zealand Plant Protection*, **53**, 258-261.

Bourdôt, G.W., Leathwick, D.M., Hurrell, G.A., & Saville, D.J. (2015) Competitive exclusion of *Cirsium arvense* in pasture: a simulated neighbour grazing-height experiment. *New Zealand Journal of Agricultural Research*, **58**, 1-12.

Cripps, M.G., Edwards, G.R., Bourdôt, G.W., Saville, D.J., Hintz, H.L., & Fowler, S.V. (2010) Effects of pasture competition and specialist herbivory on the performance of *Cirsium arvense*. *Biocontrol Science and Technology*, **20**, 641-656.

Cripps, M.G., Edwards, G.R., Bourdôt, G.W., Saville, D.J., Hinz, H.L., & Fowler, S.V. (2011) Influence of insects and fungal pathogens on individual and populations parameters of *Cirsium arvense* in its native and introduced ranges. *Biological Invasions*, **13**, 2739-2754.

Eaton, J.W., Bateman, D., & Hauberg, S. (2009) *GNU Octave version 3.0.1 manual: a high-level interactive language for numerical computations* CreateSpace Independent Publishing Platform.

Leathwick, D.M. & Bourdôt, G.W. (2012) A conceptual model for the population dynamics of *Cirsium arvense* in a New Zealand pasture. *New Zealand Journal of Agricultural Research*, **55**, 371-384.

Leathwick, D.M., Bourdôt, G.W., Hurrell, G.A., & Saville, D.J. (2006) Intraspecific plant density effects in *Cirsium arvense*. *New Zealand Journal of Agricultural Research*, **49**, 13-24.

**Table 1.** Calculating $c_{r}^{t}$, recruitment of aerial shoots from old roots (month^-1^) using aerial shoot census data for *Cirsium arvense* from the control plots (not treated with a bio-herbicide) as described in Bourdôt *et al.* (1995). The initial census date was Oct 13^th^ 1991.

| Date | Month | Number of shoots m^-2^. averaged across 3 untreated sites and 2 replicates | Estimated old root dry mass (g m^-2^) from [Bourdôt *et al.* (2006](#_ENREF_2)) | Aerial shoot births m^-2^ | Aerial shoot births m^-2^ per month | Aerial shoots formed per month per g of old root dry mass |
| --- | --- | --- | --- | --- | --- | --- |
| 21/09/1991 | 0.00 |  | 76.8 |  |  |  |
| 13/10/1991 | 0.73 | 43 | 65.8 |  |  |  |
| 19/10/1991 | 0.93 | 43 | 63.1 | 0 | 0.00 | 0 |
| 2/11/1991 | 1.40 | 46 | 57.2 | 4 | 8.57 | 0.15 |
| 17/11/1991 | 1.90 | 42 | 51.5 | 2 | 4.00 | 0.08 |
| 1/12/1991 | 2.37 | 42 | 46.6 | 3 | 6.43 | 0.14 |
| 16/12/1991 | 2.87 | 35 | 42.0 | 2 | 4.00 | 0.10 |
| 8/01/1992 | 3.63 | 40 | 35.7 | 9 | 11.74 | 0.33 |
| 23/01/1992 | 4.13 | 38 | 32.1 | 5 | 10.00 | 0.31 |
|  |  |  |  |  | Average $c_{r}^{t}$ | 0.16 |
|  |  |  |  |  | Standard deviation | 0.12 |

Fig. 1. Data for the height of the aerial shoots of *Cirsium arvense* (circles) as measured in different months of the year in two populations of the weed in pasture in New Zealand in the absence of control operations (Figs. 3 A & B in Cripps *et al.* (2011)) and the corresponding least squares fitted curve (solid line, equation 2).
